# Data analysis of ambient intelligence in a healthcare simulation system: a pilot study in high-end health screening process improvement

**DOI:** 10.1186/s12913-021-06949-5

**Published:** 2021-09-08

**Authors:** Ming-Shu Chen, Kun-Chih Wu, Yu-Ling Tsai, Bernard C. Jiang

**Affiliations:** 1 Department of Healthcare Administration, College of Management and Healthcare, Asia Eastern University of Science and Technology, No.58, Sec. 2, Sihchuan Rd., Pan-Chiao Dist., New Taipei, 22061 Taiwan, Republic of China; 2 Department of Management Center, National Chung-Shan Institute of Science & Technology, Taoyuan City, 32546 Taiwan, Republic of China; 3 Department of Industrial Management, National Taiwan University of Science and Technology, Taipei City, 10607 Taiwan, Republic of China

**Keywords:** Health screening, Healthcare simulation system, Process improvement, Service quality

## Abstract

**Background:**

This study aimed to reduce the total waiting time for high-end health screening processes.

**Method:**

The subjects of this study were recruited from a health screening center in a tertiary hospital in northern Taiwan from September 2016 to February 2017, where a total of 2342 high-end customers participated. Three policies were adopted for the simulation.

**Results:**

The first policy presented a predetermined proportion of customer types, in which the total waiting time was increased from 72.29 to 83.04 mins. The second policy was based on increased bottleneck resources, which provided significant improvement, decreasing the total waiting time from 72.29 to 28.39 mins. However, this policy also dramatically increased the cost while lowering the utilization of this health screening center. The third policy was adjusting customer arrival times, which significantly reduced the waiting time—with the total waiting time reduced from 72.29 to 55.02 mins. Although the waiting time of this policy was slightly longer than that of the second policy, the additional cost was much lower.

**Conclusions:**

Scheduled arrival intervals could help reduce customer waiting time in the health screening department based on the “first in, first out” rule. The simulation model of this study could be utilized, and the parameters could be modified to comply with different health screening centers to improve processes and service quality.

## Background

With the rapid developments in medical technology, the economy, and increases in average incomes, the general public’s average life expectancies have increased. As a result, middle-aged individuals are paying more attention to preventive healthcare and health screening. Recent statistics have shown that the average life expectancy of Taiwanese has reached 80.69 years old (National Development Center, Taiwan Population Estimate, 2022) [1], officially entering the WHO-defined aged nation. The average life expectancy has increased year by year for the last 10 years and is 3 years more than in 2006 (Department of Statistics, Ministry of the Interior, 2019) [2]. By 2026, the proportion of the population aged 65 and over in Taiwan will reach 21%, making Taiwan a super-aged society [3]. In addition, the high mortality rate caused by malignant tumors has led to the growth of health concerns. Malignant tumors are the top cause of death in Taiwan; patients dying of malignant tumors accounted for 28.2% of the total number of deaths in 2018—an increase of 1.6% compared with that in 2017 (Ministry of Health and Welfare, 2019) [4]. Nevertheless, the early identification of malignant tumors paired with suitable treatment greatly increases the probability of survival. This fact has also given rise to more attention being paid to preventive health screening.

With the improvement of economic conditions, the public has gradually attached importance to healthcare service quality. In recent years, various large-scale hospitals and professional health screening centers have purchased expensive medical instruments and devices to provide high-end health screening services. Higher-end health screenings are more expensive self-pay health checks that have been recently prevalent in Japan, South Korea, and Taiwan, including endoscopy and imaging check-ups. In Asia—including Japan, South Korea, Singapore, Taiwan, and even modern Chinese cities such as Beijing, Shanghai, or Hong Kong—high-end health screening, according to a central design concept, emphasizes that all kinds of precision instruments must be inspected in the same unit or department. As a result, this demonstrated one or a half-day high-efficiency service. The entire inspection process includes Europe, the United States, and other places less covered gastrointestinal endoscopy with anesthesia and high-end imaging examinations (as shown in Table 3, including gastrointestinal endoscopy with anesthesia [L], Endoscopic anesthesia recovery [M]; and Low-dose computed tomography of the lung screening [K], coronary artery [U]). Therefore, the specific contributions of this study have practical reference value, simulated parameters, and reduced waiting time through effective strategies for advanced Asian cities with high-end health screening. The results of this study can be used as a reference for relevant hospital units.

Moreover, the main contribution of this paper is to expand the scope of research to a complicated process of an entire health care department. While most of the existing literature on health care simulation focuses on one or few examination units, this paper takes into account the whole process of screening packages. Several doctors, nurses, and customers were involved in the system—hence, the increased difficulty in data collection and pre-processing. As many examination units were considered, the constraints and limitations were also increased. Furthermore, we present three improvement strategies different from those used in the literature. In addition, many centers have introduced smart health screening environments, which not only meet the needs of the market but also satisfy the customers’ expectations towards high-end health screenings. With the high maturity and widespread application of ambient intelligence (AmI), its application in healthcare has also been increasing to meet critical patient needs. The idea of AmI in healthcare is to create a digital environment that provides intelligent technologies with sensitive, adaptive, and responsive tools to empower physicians and patients [5]. AmI includes technologies such as sensors and devices interconnected through a network, as well as software development for recognition, reasoning, and analyzing collected data [6].

In order to improve the mixed registration of an orthopedic outpatient clinic, Lu et al. used a simulation method to reduce outpatient clinic waiting times, improving patients’ waiting times of on-site outpatient department (OPD) registration by 29–36% and the waiting time for appointment scheduling by 61–63%, which significantly improved the patients’ service satisfaction [7]. Weng et al. used simulation data to evaluate the potential bottlenecks in the emergency room (ER), improve patient flow, and reduce ER waiting times [8]. Moreover, a simulation model can also be used to evaluate the different scenarios of nursing personnel and be modified to create the most appropriate operating model. Wong et al. randomly simulated patient flow and surgical data to improve the service quality and efficiency of the ER. Improving medical quality and reducing patients’ waiting times to further improve satisfaction has been an important and critical indicator in hospital management decision-making [9].

Most previous studies used discrete event simulation (DES) technologies to solve problems such as the reduced patient satisfaction caused by excessive waiting times, low utilization rate of expensive medical instruments and devices, or the lack of physicians caused by inefficient scheduling systems. As a randomized system, simulation has been comprehensively applied to many industries, where both the manufacturing and service industries use simulation as a research tool. In recent years, numerous studies have started to use simulation software to improve or optimize medical service processes. Further, the research scope has grown to include human resource allocation, outpatient or ward process planning, ER process planning, and bed or instrument facility planning [10, 11].

Among the several studies on modeling DES in a healthcare system, simulations in emergency departments are the most popular topic. Considering that ER is the busiest and most crowded hospital area, improving ER-allocated resources might be the most pressing problem. Vanbrabant et al. reviewed the literature of ER operations from the field of operations research and provided a classification in terms of key performance indicators (KPIs) and improvement strategies [12]. Wang et al. performed a case study of an ER in a hospital in France, applying the ARIS Toolset to analyze the patient visit process and the arena software to model the patient flow [13]. Meanwhile, Zhao et al. applied the DES model to detect ER bottlenecks, used benchmarks to set an achievable target, and conducted process improvements according to the design of experiment (DOE) technique [14].

Zeinali et al. reconfigured resource allocation through a simulation system to reduce congestion and improve patient flow in the ER [15]. The critical requirements for the simulation model were the frequency of each route, patient arrival rate, and service duration. Kuo et al. analyzed the patient flow of an ER and developed a DES model considering different policies such as increasing resources, reallocating resources, and using staggered shifts [16]. On the other hand, Aboueljinane et al. used a DES model to analyze emergency services and the performance of referring patients to an adequate care institution when necessary to improve emergency medical services and the optional efficiency of emergency medicine [17]. Venkatadri et al. used a DES model to make process improvements in the cardiac catheterization room and reduce the waiting times and turnaround times of outpatients and inpatients [18].

Furthermore, Hahn-Goldberg et al. applied the DES model to analyze the sufficiency of the Neurovascular Unit at Toronto Western Hospital in Toronto, Canada [19]. In order to perform further analysis of the department’s capacity, they applied the Simul8 discrete event simulation software based on actual patient flow data. Their result confirmed that 20 beds would be able to accommodate the demand and showed that there was still a 20% growth in the number of patients under the current capacity. Shi et al. used two measurement indicators: “how can a clinic effectively utilize its resources?” and “how long does a patient need to wait?”; and a multifactorial experimental design to investigate the effects of six system performance parameters (arrival rate, non-display rate, delivery rate, new patient rate, number of repeated appointments, and nurse appointment rate) [20].

In addition, Rahman et al. used the FlexSim software to simulate the different types of patients at cancer outpatient clinics and reduce the total examination time spent by patients at a clinic [21]. On the other hand, Brenner et al. used SIMUL8 software to analyze the data of service processes and flows in the ER to simulate and calculate the total service volume of ER patients [22].

## Methods

### Materials

**Fig. 1:**
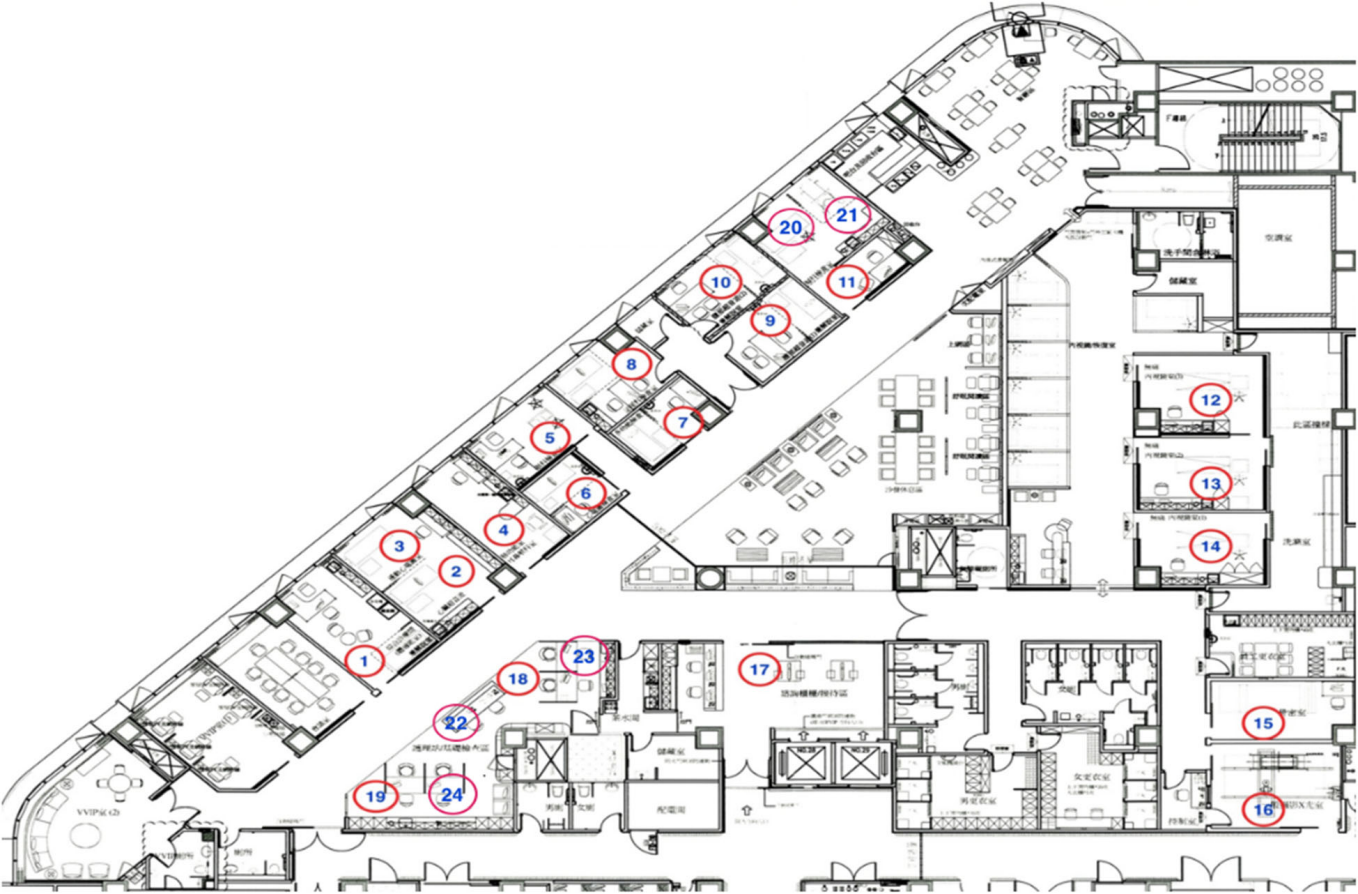
High-end health screening space floor plan and room number

This study was conducted in a health screening center at a tertiary hospital in northern Taiwan from September 2016 to February 2017. Customers at the health screening center spent at least USD 800 on high-end health screening packages. Data, including the waiting time spent at each examination station, were collected on 2342 customers. There were 112 working days in the abovementioned research period after excluding weekends and national holidays. Therefore, an average of 21 customers received high-end health screenings each day. The spaces used for examinations included examination rooms 1 ~ 24, as shown in Fig. 1 and Table 1.

**Table 1:** Names of various examination rooms on the health screening space floor plan

Room No.	Space Name	Room No.	Space Name
1	Comprehensive Examination Room	15	Bone Density Examination Room
2, 3	Cardiac Ultrasound Room	16	X-ray Examination Room
4	Otolaryngology Room	17	Information/Consultation Station
5	Ophthalmology Examination Room	18	Vision and hearing Examination
6	ECG Room	19	Questionnaire Completion Station
7	Multifunction Examination Room	20, 21	Gynecology Examination Room
8	General Examination Room	22	Nursing Station
9,10	Abdominal Ultrasound Room	23	Blood Pressure Measurement
11	Gynecology Examination Room	24	Blood Drawing Station
12, 13, 14	Endoscopy Examination Room		

This study aimed to reduce waiting times and improve the utilization rate of medical facilities and physicians and/or nursing manpower. The customers were classified into six types, as summarized in Table 2, according to the health examination package they had selected. The packages for the high-end health examination offered at Far Eastern Memorial Hospital (FEMH) were designed for customers across genders and ages. The first three types were for female customers, and the last three types were for male customers. In addition, customer types *X*_1_ and *Y*_1_ were used to represent customers over the age of 55. On the other hand, customer types *X*_2_ and *Y*_2_ were used to represent customers under 55. Meanwhile, the remaining types were regardless of age. Customers were divided into two groups based on the age of 55 because part of the customers was younger females, which were from a company executive’s client, having a lower risk of disease, and a smaller breast among young Asian women. While we would usually prioritize “breast ultrasound” in the examination [O] check-up, we recommend the “monograph” check-up for customers older than 55 years old. In the last column of Table 2, the order of the optimized health examination process—based on team members’ past experiences and the joint discussions of the internal physician and nursing experts—is shown. These health examination processes are given by practitioners based on best practices and reference processes. Customers who chose different health examination program sets or had deviant pathways were excluded in this study. Furthermore, the six customer types are the major health examination program sets in the selected health screening center. The names and codes of various examination items are shown in Table 3, where examination [O] and examination [S] represents female customers and examination [T] represents male customers. Compared to other customer types, *X*_3_ and *Y*_3_ received additional examination [U].

**Table 2:** Examination processes of six customer types

Type	Sex	Age	Health Examination Process
*X*_1_	Female	> 55	*A* → *B* → *C* → *D* → *E* → *F* → *G* → *H* → *I* → *J* → *K* → *L* → *M* → *N* → *O* → *P* → *Q* → *R* → *S* → *V.*
*X*_2_	Female	< 55	*A* → *B* → *D* → *E* → *F* → *G* → *C* → *H* → *I* → *L* → *M* → *J* → *P* → *K* → *N* → *O* → *R* → *Q* → *S* → *V.*
*X*_3_	Female	all	*A* → *G* → *B* → *D* → *F* → *E* → *C* → *H* → *I* → *J* → *K* → *U* → *L* → *M* → *P* → *N* → *O* → *S* → *R* → *Q* → *V.*
*Y*_1_	Male	> 55	*A* → *B* → *C* → *D* → *E* → *F* → *G* → *H* → *I* → *K* → *J* → *L* → *M* → *N* → *P* → *Q* → *R* → *T* → *V.*
*Y*_2_	Male	< 55	*A* → *B* → *E* → *F* → *G* → *D* → *C* → *K* → *H* → *I* → *J* → *L* → *M* → *N* → *R* → *P* → *Q* → *T* → *V.*
*Y*_3_	Male	all	*A* → *G* → *B* → *C* → *F* → *D* → *E* → *H* → *I* → *J* → *K* → *U* → *N* → *L* → *M* → *P* → *T* → *R* → *Q* → *V.*

**Table 3:** Names and codes of various examination items including simulation data distributions and expressions

Code	Examination Items (min)	Data distribution	Expressions
A	Check-in at the front desk	NA	NA
B	Changing health examination clothes (dressing)	Uniform	UNIF (5,10)
C	Confirmation of data, completion of a questionnaire	Uniform	UNIF (15,20)
D	Basic measurement of height and weight	Erlang	1 + ERLA (1.23,2)
E	Vision and hearing	Erlang	0.999 + ERLA (0.546,2)
F	Measurement of blood pressure	Exponential	0.999 + EXPO (0.626)
G	Blood drawing	Erlang	0.999 + ERLA (1.06,3)
H	ECG examination	Lognormal	1 + LOGN (3.42,1.37)
I	Abdominal ultrasound	Lognormal	1 + LOGN (2.88,2.52)
J	Cardiac ultrasound	Gamma	1 + GAMM (1.41,5.12)
K	Low-dose computed tomography of the lung screening	Uniform	UNIF (5,10)
L	Gastrointestinal endoscopy with anesthesia	Uniform	UNIF (15,30)
M	Endoscopic anesthesia recovery	Uniform	UNIF (15,20)
N	Thyroid ultrasound	Lognormal	1 + LOGN (4.51,3.04)
O	Female breast ultrasound or monograph	Beta	0.999 + 29^a^BETA (1.35,2.55)
P	Lunch	Uniform	UNIF (10,15)
Q	Consultation with ophthalmologist	Lognormal	1 + LOGN (1.99,1.64)
R	Consultation with otolaryngologist	Lognormal	1 + LOGN (3.64,3.16)
S	Consultation with gynecologist and obstetrician	Erlang	1 + ERLA (1.2,4)
T	Consultation with urologist	Lognormal	1 + LOGN (4.51,2.66)
U	Low-dose computed tomography of the coronary artery	Uniform	UNIF (20,30)
V	Leaving the medical center after completing the examinations	NA	NA

Data Distribution and Expression including, Uniform: UNIF (*a*, *b*); Erlang: ERLA ($\frac{1}{\lambda },k$); Exponential: EXPO ($\frac{1}{\lambda }$); Lognormal: LOGN (*μ*, *σ*); Beta: BETA (*β*, *α*); Gamma: GAMM ($\frac{1}{\lambda },\alpha$)

Table 2 shows the actual process of six customer types. In this actual process model of the simulated flowchart, there were more limitations on the health screening process, unlike other single examination units or departments, including which check-up station should be in front and at the back. For example, the first four items must be prioritized before other examinations. The customers should first finish the check-in [A], dressing [B], questionnaire filling [C], and basic measurements [D]. Then, the customers will be different before and after completing the basic measurement [D]. Subsequently, the customers will undergo different procedures. Some customers will first undergo blood drawing [G], blood pressure [F], vision and hearing [E], and basic measurements [D], while others will first undergo basic measurements [D], vision and hearing [E], blood pressure [F], and blood drawing [G]. Other examinations can be arranged according to the set route (Table 2). In addition, there are a number of fasting examinations that must be completed on an empty stomach, including blood drawing [G], abdominal ultrasounds [I], and gastroenterological examination and recovery [L to M]. These checks must be completed before lunch [P] and [I] must be arranged before [L] and [M], but other examinations can be arranged during the waiting process.

#### Data collection

This preliminary study on the application of AmI analyzed the data collected from smart wristbands, combining RFID and a Wi-Fi tag worn by customers receiving health screenings. When the customers checked in, the front desk staff would first collect their basic information. After the customers connect their wristbands to the RFID sensor at the clinic’s door, the system could then automatically recognize their identity and input the time. The sensor would sense their wristbands when subjects enter the clinic and exit after an examination. Therefore, the data on the time spent in the examination could be collected.

Subsequent to filtering and screening the data, this study obtained the complete and actual data needed to calculate the time spent by customers on each examination item. The screening limitations included: (1) screening according to the six customer types and hypothetical conditions; (2) deletion of records when customers missed the sensor while either entering or exiting the clinic room; and (3) deletion of extreme values due to examination times exceeding two times the mean. The data were collected by RFID and Wi-Fi tags, and each examination item was analyzed through input analytics for their data distributions and the parameters used for services, as shown in Table 3. It is evident that besides the lunch time [P], Gastrointestinal endoscopy with anesthesia [L], Endoscopic anesthesia recovery [M], and LDCT of the coronary artery [U]; and Confirmation of data, completion of a questionnaire [C] need adequate time.

For the six customer types screened in this study, there were a total of 2342 valid samples. The data analysis results indicate that the six types of distribution ratios are as follows: X1 (10.93%), X2 (29.89%), X3 (1.58%): Y1 (20.11%), Y2 (34.03%), and Y3 (3.465). According to the statistics of the customers’ arrival time and the distribution of the number of customers, only 16 customers checked in before 7:00 AM. On the other hand, most customers (1222 in total) checked between 7:00 and 8:00 AM. The number of customers checked in before 8:00 AM accounted for 52.2%, followed by a total of 877 customers checked in between 8:00 and 9:00 AM (37.4%). Customers’ daily arrival times were concentrated between 7:30 and 8:30 in the morning.

### Methods

This study used Arena simulation software (ARENA 14.5, 2016) to analyze the collected data, including the actual time spent at each examination station and the manpower required. Furthermore, it employed RFIDs and Wi-Fi tag wristbands to collect the data of six customer types and the health examination process to develop a model conforming to the current status of the health screening center in this study.

Moreover, this study implemented different improvement strategies and proposed various system simulation models to evaluate whether the waiting time could be reduced and whether the utilization rate of medical resources could be improved. Few studies have investigated the application of simulation systems to improve the scheduling and waiting times of high-end health screenings. The health screening process is different from other industrial process improvement simulations—it could be more complex, require more restrictions, and require more parameters to regulate. In this study, we provided the original approach on high-end health screenings in the modeling approach, including various examination items, data distributions, and parameter expression setting suggestions (as in Tables 3 and 4). This study could serve as a pilot study to facilitate the subsequent development of relevant studies.

**Table 4:** Real data distributions; expressions and statistical analysis of various examination items

Code	Each Examination Items (min)	Simulation		Real data			95% CI	
		Mean	Stds Dev.	N	Mean	Std. Dev.	Up	Low
D	Basic measurement of height and weight	3.46	2.22	1957	3.47	1.80	3.55	3.39
E	Vision and hearing	2.09	1.48	1821	2.09	1.29	2.15	2.03
F	Measurement of blood pressure	1.63	0.63	1863	1.63	1.14	1.68	1.58
G	Blood drawing	4.18	3.09	1565	4.18	2.34	4.30	4.06
H	ECG examination	4.42	1.37	1305	4.45	1.65	4.54	4.36
I	Abdominal ultrasound	3.88	2.52	1412	3.82	2.10	3.93	3.71
J	Cardiac ultrasound	8.22	3.20	1106	8.23	3.21	8.42	8.04
L	Gastrointestinal endoscopy with anesthesia^a^	22.5	18.75	267	26.24	13.47	27.86	24.62
N	Thyroid ultrasound	5.51	3.04	727	5.45	2.92	5.66	5.24
O	Female breast ultrasound or monograph	11.04	6.23	715	10.96	6.39	11.43	10.49
Q	Consultation with ophthalmologist	2.99	1.64	311	2.99	1.93	3.20	2.78
R	Consultation with otolaryngologist	4.64	3.16	533	4.64	3.09	4.90	4.38
S	Consultation with gynecologist and obstetrician	4.80	4.38	350	5.82	2.46	6.08	5.56
T	Consultation with urologist	4.59	2.66	255	5.46	2.49	5.77	5.15

^a^It means the simulation mean did not lie within the confidence interval range in real data—it is the [L] station

#### Simulation assumptions

In consideration of the examination requirements and the limitations of the examination space and items of the health screening center (see Table 1), a number of assumptions were made for the health screening simulation: (1) there are at least 30 customers scheduled each day; (2) the patients or customers allocation at the waiting area is simulated according to the FIFO rule (first in, first out); (3) the ophthalmology examination room (No. 5) is a shared space, where basic examination item D is performed before 12 PM and examination item E after 12 PM; (4) one of the abdominal ultrasound rooms (No. 9) is a shared space, where male customers only receive thyroid ultrasound (examination item N), while female customers receive both examination items I and N; and (5) one of the abdominal ultrasound rooms (No. 10) is a shared space, where examination item *I* w is performed before 12 PM, and male customers receive examination item *T* after 12 PM.

#### Simulation process limitations

There are scanty studies on the simulation of health screening processes because there are numerous simulation limitations, insufficient actual data, dependency, and cause-and-effect relationships among different examination items. The limitations of the simulated health screening process in this study are as follows: (1) the customers needed to fast before the blood test, abdominal ultrasound, or endoscopy examinations; (2) blood test, ECG, abdominal ultrasounds, cardiac ultrasounds, and lung cancer screening needed to be performed before endoscopic examinations; (3) abdominal ultrasounds needed to be performed before cardiac ultrasounds; (4) female customers needed to concurrently receive thyroid ultrasound and breast ultrasounds during ultrasound examinations, as well as receive gynecology ultrasounds and new pap smears at the gynecology and obstetrics station; (5) male customers needed to receive prostate ultrasounds at the urology station; and (6) the computed tomography for the coronary artery needed to be scheduled the day before, as it requires an expensive medical device. Therefore, most of the hospital radiology information system (RIS) has set the previous day before the advanced diagnostic imaging procedures (CT/MRI) scheduling. The scheduling order must be in accordance with the original schedule, and it could not temporarily change the scheduling. Based on the module setting of the constructed model, this study developed a simulation model conforming to the study site, where the study design was based on a model to divide individuals into different types of customers entering the system at the same time point.

#### Model development

Considering the current status of the subjects and the conditions of various examination items, this study used the actual time data collected from the subjects to develop the simulation model for the health screening processes of the six customer types. Because the service hours of the screening center in this study started at 7:00 AM upon check-in until the completion of the health screening process, which is around 4:00 PM, the operating hours set in the simulation was 9 hours. The total number of replications was set at 50 times, and the time unit of the simulation was set as minutes.

The research team includes industrial engineering (IE) experts, clinical professionals, and related attending physicians, health screening-related professional teams. The IE experts including three Ph.D., 2 were university professors, 1 was a national research unit researcher, specializing in healthcare administration, hospital process improvement experts, operational research, and human engineering. The process improvement team consisted of 16 professionals (2 cardiologists, a nephrologist, a gastroenterologist, a rehabilitation specialist, an administrator, 3 nurses, 1 gastroenteric technician, 2 health managers, and 4 major IE experts) from 8 domains. The team participated in discussion meetings where different professionals provided suggestions based on their different perspectives.

The results showed that the real parameters were within the 95% confidence interval of the output parameters from the model. Because the examination items [B], [M], and [P] did not ask to collect the data, there were no real data from RFIDs and Wi-Fi tag wristbands to analyze; it fitted the range of the practitioner’s experience perception. In addition, possibly because the [K] & [U] check-ups combine inspections with other outpatient units, both were excluded. The statistical results from the other examination stations used actual data from different subjects. The relevant data contained a 95% confidence interval compared with the simulation mean and standard deviations, as shown in Table 4. Due to the bias of the fitting distribution, the simulation mean of [L] did not lie within the confidence interval range, most probably because the analysis tool could not provide an adequate distribution to fit the true distribution. The parameters shown in the table were from the best fitting distribution of the candidate distributions.

**Fig. 2:**
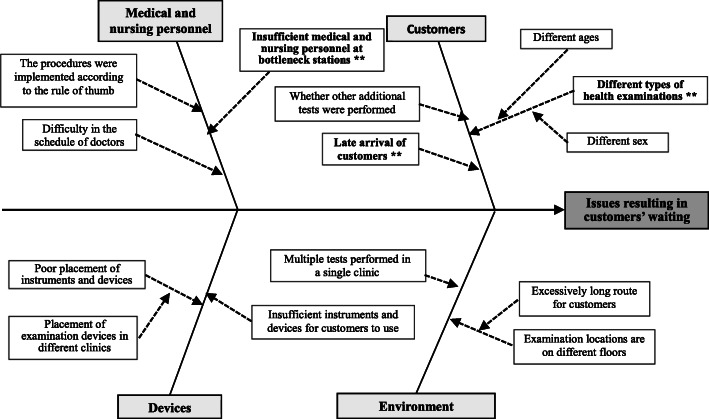
Cause and effect diagram of causes resulting in prolonged waiting times

In order to develop simulation strategies, this study first analyzed the potential main causes of the prolonged waiting time. This study plotted a cause-and-effect diagram (Fig. 2) to determine several important factors (marked as **) resulting in prolonged waiting times to investigate further the main causes of the prolonged waiting times of customers and formulate improvement measures, after full discussions with IE experts, physicians, nurses, and administers based on the Team Resource Management (TRM) methodology [23] and the suggestions provided by professional process improvement teams. The factors included the late arrival of customers, insufficient medical and nursing manpower at bottleneck stations, and different types of health examinations. Therefore, this study developed three improvement strategies for simulation: (1) adjusting the mixture of customer types; (2) increasing human resources at bottleneck stations; and (3) adjusting customer arrival times.

#### Model validation

Verification and validation tests were conducted before taking the effort on the experimental phase to ensure that the baseline model could accurately and relevantly represent the actual system [24]. The validation process was performed. Firstly, the model was essentially verified by checking the operational logic and the experimental conditions. The animation and the debugger tool were also adopted to trace the entities of different types. Then, the face validation was performed to validate the simulation result of the baseline model by consulting the clinical experts and health screening managers.

Finally, the baseline model was validated by comparing the simulation outputs to those of the real system. Additionally, the simulation model was developed according to the processing flow and the examination time of the real system. Hence, the outputs of the simulation results should resemble the real one if the mode is built right. Table 5 shows the comparison results between the real data and the simulation results, in which the simulation results were obtained by taking the average of 50 replications.

**Table 5:** Comparison of the real data for average waiting time and total examination time of customers to the simulation results

Customer Type	Real-data			Simulation		
	Number Out (#)	Waiting Time (min)	Total Time (min)	Number Out (#)	Waiting Time (min)	Total Time (min)
*X*_1_	2.30	61.22	192.16	2.36	61.24	192.06
*X*_2_	6.28	49.64	186.24	6.34	49.53	186.37
*X*_3_	0.33	20.39	64.42	0.36	20.37	63.96
*Y*_1_	4.22	66.98	194.20	4.40	66.60	194.41
*Y*_2_	7.15	66.24	197.53	7.64	66.67	197.99
*Y*_3_	0.73	49.21	138.78	0.86	48.89	139.20

The three factors are imposed to validate the outputs, as shown in Table 5. Number Out represents the number of customers receiving this health examination type who finished and exited the process. Waiting Time denotes the individuals’ waiting time, while Total Time denotes the total time spent by individuals in the process. The output data of Waiting Time and Total Time were the time accumulated for a customer from the entering to the exiting process. Table 5 shows that the simulation results were quite close to the real data.

## Results

The simulation results are summarized in the following tables. The data of the average waiting time and total examination time of the simulated health screening customers are shown in Table 5. The simulation results showed that the average waiting time of the X3 module was the shortest (20.37 mins). Overall, the average waiting time and total examination time of the male customers were longer than those of the female customers. The waiting time data of the examination items at various examination stations are shown in Table 3, while the data of Number Out are shown in Table 5. As shown in Table 6, this study found that gastrointestinal endoscopy [L] was the major bottleneck examination station, followed by stations [J] and [K].

**Table 6:** Current status results at the waiting area of various examination items in the system simulation

Queue	Waiting time (min)	Number Waiting
Height, weight [D]	0.1126	0.0055
Vision and hearing [E]	0.0971	0.0047
Blood pressure measurement [F]	0.0985	0.0048
Blood Drawing [G]	0.1058	0.0051
ECG [H]	0.0804	0.0038
Abdominal ultrasound [I]	0.0507	0.0024
Cardiac ultrasound [J]^a^	^a^15.4203	0.6852
Low-dose CT scan of the lungs [K]^a^	^a^8.7374	0.3952
Coronary angiography [U]	0.5673	0.0033
Gastrointestinal endoscopy [L]^a^	^a^35.2242	^a^1.5535
Thyroid ultrasound [N] (Male)	0.0338	0.0008
Thyroid ultrasound [N] (Female) Plus female breast ultrasound [O]	0.0335	0.0007
Consultation at Division of Ophthalmology [Q]	0.3249	0.0139
Consultation at Division of Otolaryngology [R]	1.4155	0.0597
Consultation at Division of Gynecology and Obstetrics [S]	0.5135	0.0096
Consultation of Division of Urology [T]	0.5854	0.0146

^a^denotes that the waiting time exceeded 5 minutes and that the examination stations were regarded as bottleneck stations

Based on the above simulation results (Tables 5 and 6) and the causes of prolonged waiting time inferred from the cause-and-effect diagram (Fig. 2), this study simulated six customer modules. An average of 21.22 customers received health examinations every day. However, meeting the expectations of the study site (the hospital) to provide services to at least 30 customers every day and under the premise that the manpower at the examination stations remains unchanged, the simulation results showed that the average waiting time was increased from 52.22 min for 21 customers to 72.29 min for 30 customers. In order to reduce the waiting time, this study developed the following three improvement strategies for the system simulation, including (1) adjusting the mixture of customer types; (2) increasing bottleneck (gastrointestinal endoscopy exam [L]) resources; and (3) adjusting customer arrival times. The actual simulation results of the three improvement strategies are shown below.

In the simulation, the examination capacity was changed from 21 to 30 customers, and three policies were compared. The first policy was based on a predetermined proportion of customer types. Compared to the current state, the average waiting time was reduced for four customer types, while the waiting time increased for the other two types. According to the simulation results, strategy 1 showed poor results, as the total waiting time increased from 72.29 to 83.04 mins. On the other hand, strategies 2 and 3 effectively resulted in lower waiting times. The total waiting time was reduced from 72.29 to 28.39 mins for strategy 2 and 72.29 to 55.02 mins for strategy 3. As shown in Table 7, strategy 3 was the overall better solution.

**Table 7:** Average waiting time and total examination time of each improvement strategy

Improvement Strategy Increase daily customer number (from 21 to 30)	Average total waiting time (min)	Average total exam time (min)
**Baseline of original condition**	**72.29**	**188.21**
(1) Adjust the mixture of customer types	83.04	211.83
(2) Increase bottleneck [L] resources	28.39	151.21
(3) Adjust customer arrival times	55.02	171.48

According to the simulation results, while strategy 2 showed the best result, the utilization rate would decrease, and the cost would increase when resources were added to the bottleneck. Therefore, we suggested applying strategy 3 to reduce the waiting time. Follow-up research could utilize this study’s simulation model and modify the parameters to comply with different health screening centers to improve processes and service quality. Further, this study found that strategy 3 (adjusting customer arrival times) could be the best strategy to decrease the total waiting time.

## Discussion

According to Bloomberg’s ranking on health care efficiency, Taiwan is in eighth place among 56 economies [25]. Not only outpatient clinics and inpatient departments face fierce competition but also high-end health screening services. Due to this competition, many customers of high-end health screenings have started to pay attention to health examination waiting time. Waiting time has a negative association with customer satisfaction in most service systems. In the case of the healthcare system, although they may not be the major factor in customer satisfaction, long waiting times lead to low satisfaction [26]. As more of the general public are inclined to choose medical service systems with high quality and short waiting times, the requirements for hospital management should not be limited to cost reduction [27]. This study aims to solve the customer waiting problem of high-end health screening centers, hoping to identify the main bottleneck examination and improve strategy as merely reducing the waiting time may not fully satisfy the customer. Studies simulating high-end health screening processes have rarely been published in international journals. However, numerous past studies have used the Arena system to simulate cases receiving healthcare processes. England and Roberts, Lu et al., and Motola et al. focused on the outpatient operating processes of different divisions [7, 28, 29], while Weng et al. and Van et al. focused on the ER [8, 30]. On the other hand, Venkatadri et al. focused on cardiac catheterization rooms [18], while Wong et al. focused on operating room processes [9]. In addition, other studies have used various analysis software packages to simulate healthcare service processes. Rahman et al. used the FlexSim software to simulate the different types of patients at cancer outpatient clinics and reduce the total examination time spent by patients at the clinic [21]. Brenner et al. used the SIMUL8 software to analyze the data of service processes and flows at an ER to simulate and calculate the total service volume of ER patients [22].

However, there is a lack of studies taking health examination centers as the research targets, especially concerning high-end health screening processes. The reason might be that more examination items and numerous stations are involved in current high-end health screening processes; thus, the conditions and limitations are also more complicated. Further, there is a lack of relevant smart facilities in most health examination centers for the collection of relevant data, such as examination times and waiting times. Such limitations make it difficult to perform relevant studies. Chen et al. proposed a policy optimization procedure for a simulation model in which five ultrasound rooms provide eight types of ultrasound services [31]. To simplify the simulation model, they assumed that no walk-in patients are allowed and are rather seen by appointment only, that they arrive on time, and that only the service time was non-deterministic. They proposed four appointment scheduling policies, having respective parameters, such as the scheduled arrival intervals and the number of patients per arrival. Then, an optimization process based on the scatter search and tabu search methods was applied to find the near-optimal parameters for each model. Chen et al. determined the best solutions for different appointment scheduling policies using mathematical programming and simulation optimization procedures based on the ultrasound examination process of a hospital ultrasound department in Taiwan. However, the near-optimal parameters for each model were not appropriate to our process model. Our simulation process is a more complex, high-order health checking process that includes multiple ultrasounds (as shown in Table 3), including Abdominal ultrasound [I], Cardiac ultrasound [J], Thyroid ultrasound [N], and Breast ultrasound [O]). It also involves more complicated endoscopic procedures performed under anesthesia [L & M], high-end imaging [K & U], and consultations with different physicians and specialists [Q, R, S, T, and U], all of which have different parameters and different conditions. The simulation strategy of Chen et al. includes four appointment scheduling policies, namely frequent arrival, mixed patient arrival, three-section mode arrival, and irregular arrival. This study has three different simulation strategies: the first policy presenting a predetermined proportion of customer types, the second policy based on increased bottleneck resources, and the third policy based on adjusting customer arrival times. In particular, Chen et al. provided the direction of the strategy developed in the third policy of this study.

According to the simulation results of the actual data of high-end health screenings, this study proposed specific suggestions for the subsequent optimization of health examination processes, study sites (health screening centers), and future researchers interested in engaging in the simulation of a medical service process system. This study found that if the distribution of customer arrival times could be effectively controlled and late arrivals could be avoided, waiting times could be effectively reduced and the total examination time shortened. These results were different from another study in a health screening center, reducing waiting time using the Design for Six Sigma method in Taiwan [32]. Therefore, future researchers are advised to use the research model to apply multiple improvement strategies and set up parameters for the model. Multiple objectives, such as cost reductions, waiting time reductions, and decreasing the utilization rate of bottleneck station resources, could be jointly simulated to analyze their feasibility. Future researchers are advised to simulate the optimized scheduling system and use the data collected in a real-time setting to develop a database and produce advice for optimized scheduling in a real-time manner according to the dynamic changes of different customers.

Furthermore, this study has the following two limitations. First, it investigated high-end health screenings, including painless gastrointestinal endoscopy with anesthesia [L], as well as coronary artery computed tomography [U], in the health examination processes of six customer types according to their sex and age. These six processes were chosen because they could account for 80–90% of the service volume of the center’s high-end health screenings. However, other health examination processes were not analyzed. Second, as more service contents and examination items are involved in high-end health screenings, it is impossible to apply the research results to other institutions (hospitals or health screening centers) due to variations of the number of doctors, quantity of examination instruments and devices, spaces, or the number of staff. However, the research methods and simulation parameters could be provided as a reference for other institutions.

Finally, based on the above studies, this study summarized several important research findings. First, a simulation system can be effectively applied to large-scale hospitals where diversified examination items, complicated conditions and limitations, different examination plans, and types of customers receiving health examinations can be simulated. These simulation modules and study design could be applied to more medical units in hospitals, such as operating rooms. Second, the simulation results showed that, although the addition of devices or medical and nursing manpower can effectively reduce customers’ waiting times, more funds and management costs are required. If customers’ arrival times are effectively controlled or a batch check-in system is developed, the waiting times of customers receiving health examinations could be significantly improved. Third, the simulation can be applied to the pre-planning of newly established health screening centers, site expansions, the planning of original units, new high-end health screening items, or simulation planning before the addition of new health screening packages to avoid unideal manpower, facility, or resource allocations, as well as poor process planning or facility planning. Although most health screening centers have considered improving the arrival rate of on-time arrivals or developing batch flow control at check-in, there is still a lack of empirical data for reference. This study used actual waiting time data to empirically prove the effectiveness of this strategy and propose two specific plans. The results of this study could provide substantial assistance to health screening centers and demonstrated that developing a good check-in strategy could improve the waiting time and medical utilization rate of customers receiving health examinations at health examination departments.

## Conclusions

This study used FIFO to perform a simulation according to the developed model based on six improvement plans and three major improvement strategies for the waiting areas of the examination stations. The results showed that the effectiveness of adding human resources to bottleneck stations was the best (Table 7), followed by adjusting customer arrival times. Arrival time was found to be a key factor, similar to the result of Van et al. (2019), who prioritized emergency department arrivals. Triaging results in lower patient waiting times for patients in the higher priority acuity class but longer waiting times for patients in the lowest priority class, which do not require immediate care and are not subject to FIFO and first-come-first-served scheduling (FCFS).

As verified by the medical staff of the study site, the customer arrival time can effectively reduce the waiting time. However, some examinations still require longer waiting times, where hurried check-up processes might decrease customer satisfaction. This study suggests that the experimental design of follow-up studies may adopt the non-FIFO principle. To evenly allocate the waiting time for high-end health screening, this study proposed the principle of the “Best Wait and Exams Ratio (BEWR)” rule. This does not allow the current simulation software to set parameters, and perhaps different algorithms can be used to build the system. Although this study used the simulation tool to conduct optimization-simulation approaches to hospital service processes or the healthcare system, there are too many limitations and conditions. Further studies should be carried out to collect health care workers’ opinions to find the most appropriate setting parameters.

According to the simulation results, this study reached two main conclusions and five specific research contributions. First, the effect of adjusting the proportions of customer types on reducing waiting times was limited. In addition, as the number of customers differs every day, the proportions of customer types varied. Although the use of a reservation system could control the number of different types of customers making advanced reservations, the effectiveness of the simulation was not as expected. Second, adding resources to bottleneck stations and adjusting customer arrival times could effectively reduce waiting times and total examination times. Provided that the number of different types of customers making a reservation is known, properly adjusting customer arrival times could pave the way for a smoother process. If the process is still congested and waiting times are not improved, manpower allocation could be adjusted accordingly to make the service process more efficient. As adding manpower to bottleneck stations increases costs, adjusting customer arrival times may be the best choice for health screening centers to improve waiting times.

This study simulated the actual data of high-end health screenings to determine the adequate distribution of parameters and data, developed an improvement strategy model, and then simulated and analyzed the comparable data. The five specific research application contributions are as follows. First, this study is the first to apply the simulation system to simulate the comparable results of different high-end health screening processes and include more examination items for the simulation data distributions and expressions setting module. The findings not only can be referenced by hospitals in Taiwan but also by high-end health screening services in Japan and South Korea, including endoscopy and advanced imaging examination. Second, the study site was a high-end health screening center. If there is a need to expand service volume or adjust the examination processes of different health examination packages, the simulation model in this study could help evaluate and verify its adequacy. Third, the simulation model developed in this study could help identify the bottleneck stations of the service processes at various health screening centers to improve further the resources at these stations. Fourth, if there is a need to add manpower or expensive resources, such as instruments and devices, when faced with the expansion or addition of examination items, the simulation model proposed in this study could help evaluate the quantity required to be added. Lastly, when the number of customers receiving high-end health screenings every day increases, this system could help assess whether the process and arrival times and the resource allocations of the examination stations require adjustment.

Although a pilot study, this research used the rule of thumb to choose six higher-order health screening processes for simulation, and the parameters were input according to the distribution of the actual health screening center data from the study site. Therefore, the simulation results are only for reference of other health screening centers. However, the common goals pursued by the health screening centers of major hospitals and healthcare institutions are to reduce waiting times, increase the number of customers receiving health examinations, and improve customer satisfaction. Therefore, the simulation model of this study could be further modified to meet the needs of different numbers of customers in different health examination centers, including arrival times, health examination processes, examination times, and different waiting intervals. Furthermore, it could even be used to perform different improvement strategy simulations to improve the quality of overall high-end health screenings.

With the rapid development of automation technology and artificial intelligence-related facilities, the application of simulation systems by medical institutions and the health industry could create significant market potential in the future. A simulation system could provide various medical units with services, including simulations of the allocation of manpower, the placement of beds, and process planning for patients at various departments. The best improvement plans can be obtained through system simulation. Hopefully, future researchers can further develop advanced models and appropriate simulation software functions for more comprehensive applications in the medical and health industry.

## Data Availability

The data that support the findings of this study are available from Far Eastern Memorial Hospital’s Research Service, but restrictions apply to the availability of these data, which were used under license/authorization for the current study, and so are not publicly available. Additional data analyses are however available from the authors upon reasonable request and with permission of Far Eastern Memorial Hospital’s Research Service.

## Abbreviations

AmI: Ambient intelligence
OPD: Outpatient department
ER: Emergency room
DES: Discrete event simulation
KPIs: Key performance indicators
USD: United States dollar
ECG: Electrocardiography
LDCT: Low-dose computed tomography
FEMH: Far Eastern Memorial Hospital
NA: Not applicable
RFID: Radio Frequency Identification
FIFO: First in, first out
FCFS: First-come-first-served
BEWR: Best Wait and Exams Ratio
